# Response to neoadjuvant treatment among rectal cancer patients in a population-based cohort

**DOI:** 10.1007/s00384-020-03744-2

**Published:** 2020-09-19

**Authors:** Elizabeth Alwers, Lina Jansen, Jakob Kather, Efrat Amitay, Hendrik Bläker, Matthias Kloor, Katrin E. Tagscherer, Wilfried Roth, Esther Herpel, Jenny Chang-Claude, Hermann Brenner, Michael Hoffmeister

**Affiliations:** 1 Division of Clinical Epidemiology and Aging Research, German Cancer Research Center (DKFZ), Im Neuenheimer Feld 581, 69120 Heidelberg, Germany; 2 Department of Medicine III, University Hospital RWTH Aachen, Aachen, Germany; 3 Institute of Pathology, University Hospital Leipzig, Leipzig, Germany; 4 Department of Applied Tumor Biology, Institute of Pathology, University of Heidelberg, Heidelberg, Germany; 5 Institute of Pathology, University Hospital Heidelberg, Heidelberg, Germany; 6 Institute of Pathology, University Medical Center Mainz, Mainz, Germany; 7 NCT Tissue Bank, National Center for Tumor Diseases (NCT), Heidelberg, Germany; 8 Division of Cancer Epidemiology, German Cancer Research Center (DKFZ), Heidelberg, Germany; 9 Genetic Tumor Epidemiology Group, University Medical Center Hamburg-Eppendorf, Hamburg, Germany; 10 Division of Preventive Oncology, German Cancer Research Center (DKFZ), National Center for Tumor Diseases (NCT), Heidelberg, Germany; 11 German Cancer Consortium (DKTK), German Cancer Research Center (DKFZ), Heidelberg, Germany

**Keywords:** Rectal cancer, Neoadjuvant treatment, Response to treatment, Pathological complete response

## Abstract

**Background:**

In rectal cancer, prediction of tumor response and pathological complete response (pCR) to neoadjuvant treatment could contribute to refine selection of patients who might benefit from a delayed- or no-surgery approach. The aim of this study was to explore the association of clinical and molecular characteristics of rectal cancer with response to neoadjuvant treatment and to compare patient survival according to level of response.

**Methods:**

Resected rectal cancer patients were selected from a population-based cohort study. Molecular tumor markers were determined from the surgical specimen. Tumor response and pCR were defined as downstaging in T or N stage and absence of tumor cells upon pathological examination, respectively. The associations of patient and tumor characteristics with tumor response and pCR were explored, and patient survival was determined by degree of response to neoadjuvant treatment.

**Results:**

Among 1536 patients with rectal cancer, 602 (39%) received neoadjuvant treatment. Fifty-five (9%) patients presented pCR, and 239 (49%) and 250 (53%) patients showed downstaging of the T and N stages, respectively. No statistically significant associations were observed between patient or tumor characteristics and tumor response or pCR. Patients who presented any type of response to neoadjuvant treatment had significantly better cancer-specific and overall survival compared with non-responders.

**Conclusion:**

In this study, patient characteristics were not associated with response to neoadjuvant treatment, and molecular characteristics determined after surgical resection of the tumor were not predictive of pCR or tumor downstaging. Future studies should include molecular biomarkers from biopsy samples before neoadjuvant treatment.

**Electronic supplementary material:**

The online version of this article (10.1007/s00384-020-03744-2) contains supplementary material, which is available to authorized users.

## Introduction

Neoadjuvant treatment is recommended for patients with locally advanced rectal cancer, usually including chemo-/radiotherapy (nCRT) followed by surgical resection [1]. The surgical approach, including abdominoperineal resection and total mesorectal excision, yields good local control and prognosis; however, it is also associated with significant peri-operative morbidity, including bowel, sexual and urinary dysfunctions [2], and a permanent colostomy in some cases, which severely affects the patient’s quality of life [3, 4].

To spare rectal cancer patients from the morbidity caused by surgical resection, the watch-and-wait approach has been suggested [5]. This approach proposes that surgery be delayed in patients who achieve a clinical complete response (cCR: no evidence of tumor in regular clinical and radiological examinations) after receiving neoadjuvant treatment [5, 6]. Case series and cohort studies have shown benefits of a watch-and-wait approach after cCR, resulting in organ preservation and equivalent oncological outcomes as compared with those of patients who achieved pathological complete response (pCR: no evidence of residual tumor cells upon pathological examination) after radical surgery [7–9]. Optimal selection of patients who are eligible for a watch-and-wait approach continues to be challenging, given that no definitive predictors of pCR have been identified [10].

Among the established molecular markers of colorectal cancer, KRAS and TP53 mutations have been associated with lack of response to neoadjuvant treatment in observational studies with small sample sizes [11, 12], while evidence for other molecular markers is still scarce [13]. Some studies have indicated that post-surgical mutations of CRC might be representative of those present in the tumor prior to administration of nCRT [14]. The aim of this study was to compare patient and tumor characteristics of rectal cancer patients according to administration of neoadjuvant treatment. Also, we aimed to describe clinical and molecular predictors of tumor response and pCR among rectal cancer patients who received neoadjuvant treatment and describe survival among responders and non-responders.

## Methods

### Study design and patient selection

The DACHS (Darmkrebs: Chancen der Verhütung durch Screening) study is a population-based case-control study conducted since 2003 in more than twenty hospitals in the south-west of Germany that was initiated to investigate the potential of screening endoscopy in the reduction of colorectal cancer risk. In addition, patients with colorectal cancer are followed up as a cohort at 3, 5, and 10 years after diagnosis [15–17]. In this analysis, patients diagnosed with rectal cancer (ICD-10 code C20) between 2003 and 2014 were included. Baseline socio-demographic and lifestyle characteristics, as well as medical and family history, were obtained by trained interviewers using a standardized questionnaire at the time of diagnosis. Clinical and pathological characteristics were determined from medical records, pathology reports, and hospital discharge letters after surgical resection of the tumor. Information on type of therapy, comorbidities, and recurrence of disease was determined from medical reports obtained from the treating physicians. Long-term follow-up was performed at 3, 5, and 10 years after diagnosis including information on recurrence of disease. Vital status and date and cause of death were determined from population registries and death certificates issued by the health authorities.

Only patients with stage II or III rectal cancer at diagnosis who underwent surgical resection of the tumor were included in this analysis. The type of surgical resection and neoadjuvant treatment schemes were provided according to general clinical guidelines and the treating physician’s decision. Molecular tumor characterization was available for a subset of these patients (see patient selection in supplementary figure 1). Molecular markers, including KRAS mutations and CpG island methylator phenotype (CIMP) status, were determined in tumor tissue specimens after surgical resection and performed on formalin-fixed, paraffin-embedded (FFPE) samples, as previously described [15].

### Tumor response and pathological complete response

Tumor characteristics including T and N stages, grade, and histology were derived from post-surgical pathology reports. Outcomes of interest included the difference between clinical (cT, cN) and pathological (ypT, ypN) T and N stages after neoadjuvant treatment. Tumor response was defined as downstaging of T, N, or both stages among patients with available information (i.e., excluding TX, NX). pCR was defined as ypT0-N0 stage determined from pathology reports after neoadjuvant treatment.

### Patient survival

Secondary outcomes included cancer-specific survival (CSS), defined as time from diagnosis until death from rectal cancer; relapse-free survival (RFS), defined as time from diagnosis until reappearance of disease, metastases, cancer death, or death from other causes; and overall survival (OS), defined as time from diagnosis until death from any cause. The association between pCR and tumor response (downstage in T or N) with survival outcomes was studied among patients who received neoadjuvant treatment adjusting for relevant co-variates such as age, clinical stage at diagnosis, adjuvant chemotherapy, and comorbidities (Charlson comorbidity score [18, 19]). In a complementary analysis, CSS, RFS, and OS were determined independently for patients who received neoadjuvant treatment and presented response or not, and compared with patients who did not receive neoadjuvant treatment.

### Statistical analysis

Socio-demographic characteristics were described for the entire patient population and stratified by whether the patient received neoadjuvant treatment or not. Chi-square or Fisher’s exact tests were used to explore the associations between patient and tumor characteristics, and the specified outcomes of tumor response and pCR. Tumor response, expressed as the change in T and N stages, was also visually explored by means of alluvial diagrams. Cox proportional hazard models were used to estimate HR and 95% CI for the secondary survival outcomes. All statistical analyses were performed using R version 3.5.1, packages dplyr, survival, and ggalluvial [20].

## Results

### Patient and tumor characteristics

Overall, 1536 patients with resected rectal cancer were included in this analysis, of whom 602 (39%) received neoadjuvant treatment. Table 1 presents patient and tumor characteristics, overall and by administration of neoadjuvant treatment or not. Overall, a larger proportion of patients (66%) was male, and median age at diagnosis was 68 years for patients who did not receive neoadjuvant treatment and 65 years for those who did. Among patients who received neoadjuvant treatment, 90% presented with clinical T3/T4 tumors and 71% with any type of lymph node involvement (N+/N1-3). The use of neoadjuvant treatment increased by year of diagnosis: 32% of patients diagnosed between 2003 and 2006 received neoadjuvant treatment, while this proportion increased to 45% for patients diagnosed between 2011 and 2014.

**Table 1:** Rectal cancer patient and tumor characteristics by neoadjuvant treatment—*n* (%)

Variable	Category	Overall (*n* = 1536)	No neoadjuvant (*n* = 934)	Neoadjuvant (*n* = 602)
Age group	≤ 64	656 (42.7)	366 (39.2)	290 (48.2)
	65–74	520 (33.9)	315 (33.7)	205 (34.1)
	≥ 75	360 (23.4)	253 (27.1)	107 (17.8)
Gender	Female	521 (33.9)	344 (36.8)	177 (29.4)
	Male	1015 (66.1)	590 (63.2)	425 (70.6)
Comorbidity index	0	899 (58.5)	518 (55.5)	381 (63.3)
	1	287 (18.7)	179 (19.2)	108 (17.9)
	2–3	350 (22.8)	237 (25.4)	113 (18.8)
Year of diagnosis	2003–2006	514 (31.6)	351 (35.3)	163 (25.8)
	2007–2010	491 (30.2)	290 (29.2)	201 (31.8)
	2011–2014	531 (32.7)	293 (29.5)	238 (37.6)
Clinical T stage	T1	54 (6.2)	54 (15.0)	0
	T2	151 (17.4)	113 (31.4)	38 (7.5)
	T3	524 (60.3)	132 (36.7)	392 (77.0)
	T4	90 (10.4)	24 (6.7)	66 (13.0)
	TX	50 (5.8)	37 (10.3)	13 (2.6)
Clinical N stage	N0	301 (35.6)	175 (50.1)	126 (25.4)
	N+	231 (27.3)	37 (10.6)	194 (39.1)
	N1	156 (18.5)	49 (14.0)	107 (21.6)
	N2	65 (7.7)	17 (4.9)	48 (9.7)
	N3	3 (0.4)	0 (0.0)	3 (0.6)
	NX	89 (10.5)	71 (20.3)	18 (3.6)
T stage after treatment*	T0	-	-	61 (10.2)
	Tis–T1	-	208 (22.3)	33 (5.5)
	T2	-	245 (26.3)	151 (25.3)
	T3	-	424 (45.5)	327 (54.8)
	T4	-	55 (5.9)	25 (4.2)
N stage after treatment*	N0	-	571 (63.9)	404 (67.7)
	N1	-	190 (21.3)	131 (21.9)
	N2	-	132 (14.8)	62 (10.4)
Patients with available tumor marker characterization		Overall (*n* = 682)	No neoadjuvant (*n* = 470)	Neoadjuvant (*n* = 212)
KRAS mutation	Non-mut	432 (68.9)	286 (66.5)	146 (74.1)
	Mutated	195 (31.1)	144 (33.5)	51 (25.9)
CIMP status	Low/neg	613 (91.2)	419 (90.3)	194 (93.3)
	High	59 (8.8)	45 (9.7)	14 (6.7)

Totals may not add up because of missing values. Clinical T stage information unavailable for 694 patients (14% received neoadjuvant). Clinical N stage information unavailable for 718 patients (15% received neoadjuvant). Missing T and N information for 2 and 41 patients who did not receive neoadjuvant treatment, respectively. Missing KRAS status *n* = 59 (27% received neoadjuvant), and missing CIMP *n* = 10 (4 received neoadjuvant). Missing T and N stage information for 5 patients who received neoadjuvant treatment

*T and N stages were determined from pathology reports after surgery for patients who did not receive neoadjuvant treatment (pT and pN) and patients who received neoadjuvant treatment (ypT and ypN)

### Molecular tumor markers

Information on tumor molecular characteristics was available for 682 (44%) patients, of whom 212 (31%) received neoadjuvant treatment (see Table 1). KRAS mutations were found in 144 (34%) patients who did not receive neoadjuvant treatment and in 51 (26%) patients who received neoadjuvant treatment (*p* = 0.069).

### Response to neoadjuvant treatment

Among 602 patients who received neoadjuvant treatment, 61 (10%) had no evidence of residual tumor after surgical resection (ypT0), 33 (5%) had ypTis-T1 tumors, 151 (25%) had ypT2, and 327 (55%) showed ypT3 tumors. After neoadjuvant treatment, 404 (68%) patients had ypN0 stage, while 131 (22%) and 62 (10%) showed ypN1 and ypN2 diseases, respectively (see Table 1).

**Fig. 1:**
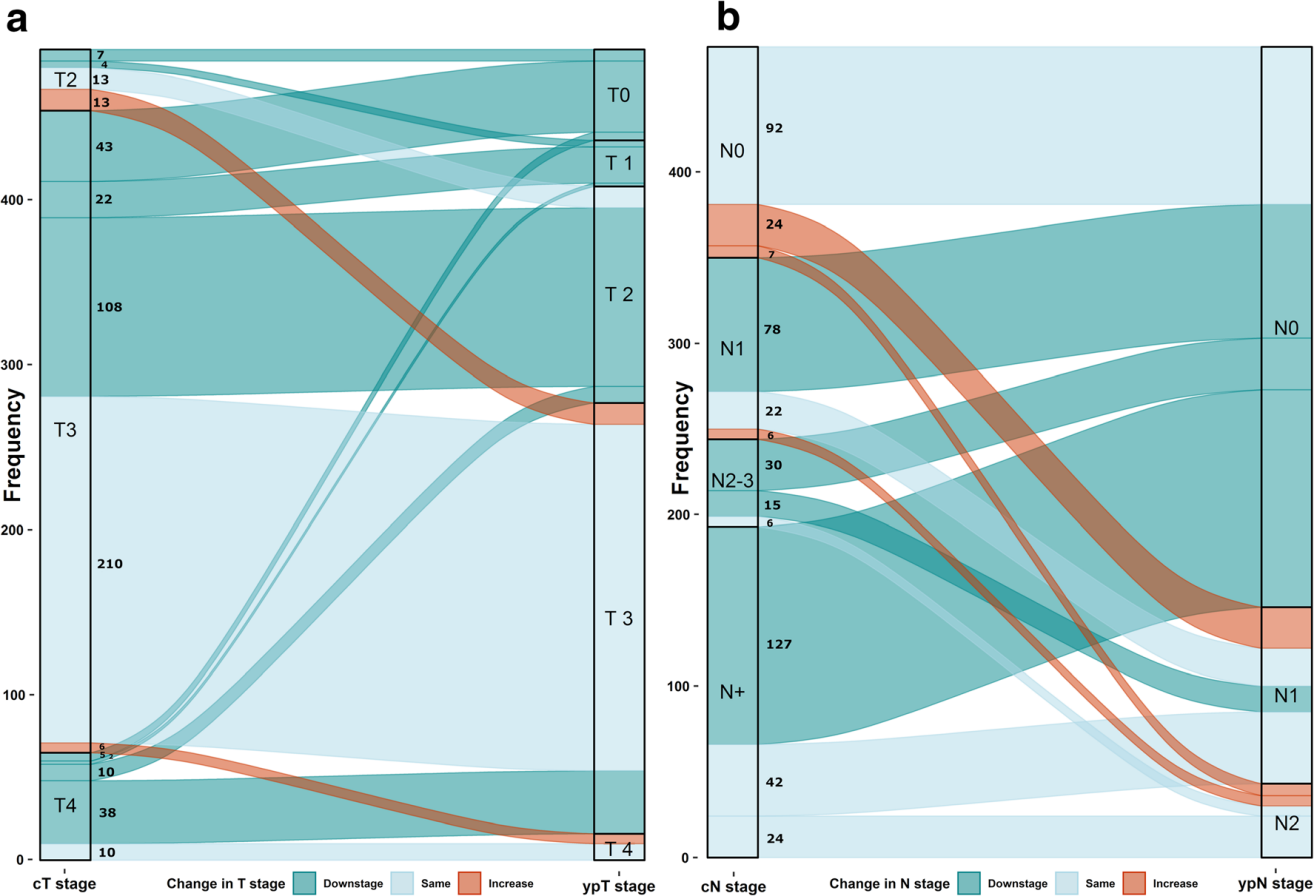
**a** Change in T stage among patients who received neoadjuvant treatment. The left column indicates the proportion of patients in each clinical T stage (cT stage) category; the right column indicates the proportion of patients in each T stage after treatment (ypT stage). **b** Change in N stage among patients who received neoadjuvant treatment. The left column indicates the proportion of patients in each clinical N stage (cN stage) category; the right column indicates the proportion of patients in each stage after treatment (ypN stage). The flow in between columns indicates the number of patients that transition between categories: dark green indicates downstaging, light blue indicates no change, and red indicates increase in stage

Table 2 presents outcomes of response to treatment among patients who received neoadjuvant therapy. pCR, defined as ypT0N0 stage after neoadjuvant treatment, was observed in 55 (9%) patients. Among patients with available information, 233 (48%) had no change in the T stage after neoadjuvant treatment, while 239 (49%) presented downstaging of the tumor, and 19 (4%) a higher T stage. Similarly, 250 (53%) patients presented a reduction in N stage, 186 (39%) no improvement, and 37 (8%) a higher N stage than was clinically assessed. The transition of patients from clinical stages (cT, cN) to pathological stages (ypT, ypN) is additionally presented in Fig. 1.

**Table 2:** Tumor response among rectal cancer patients who received neoadjuvant treatment (*n* = 602)

Outcome	Category	*n* (%)
pCR (ypT0, ypN0)	No	539 (90.7)
	Yes	55 (9.3)
Change in T stage (ypT–cT)	− 4	5 (1.0)
	− 3	45 (9.2)
	− 2	39 (7.9)
	− 1	150 (30.6)
	0	233 (47.5)
	1	19 (3.9)
Any change in T stage	Decreased	239 (48.7)
	Same	233 (47.5)
	Increased	19 (3.9)
Any change in N stage	Decreased	250 (52.9)
	Same	186 (39.3)
	Increased	37 (7.8)

*pCR*, pathological complete response (ypT0, ypN0)

Change in T stage calculated as the difference between ypT stage (determined from surgical specimen) and cT stage, for patients with available information (*n* = 491)

Table 3 presents patient and tumor marker characteristics by outcomes of pCR and tumor response among patients who received neoadjuvant treatment. No statistically significant associations were observed between the investigated characteristics and tumor response. Patient clinical characteristics did not show any relevant associations to predict pCR or tumor downstaging in univariate analyses. Patients who presented downstaging in the number of involved lymph nodes or showed any response to neoadjuvant treatment had a somewhat lower, although not significant, proportion of KRAS mutations compared with those who showed no improvement (17% vs 24%, *p* = 0.441 and 22% vs 30%, *p* = 0.379, respectively).

**Table 3:** Pathological complete response and tumor response by patient and tumor characteristics among rectal cancer patients who received neoadjuvant treatment (*n* = 602)

		pCR (ypT0, ypN0)			Downstaging in T (ypT < cT)			Downstaging in N* (ypN < cN)			Any response (ypT, ypN)		
		No	Yes	*p* value	No	Yes	*p* value	No	Yes	*p* value	No	Yes	*p* value
		*n* = 539	*n* = 55		*n* = 252	*n* = 239		*n* = 100	*n* = 250		*n* = 133	*n* = 352	
Age group	≤ 64	259 (48.1)	28 (50.9)	0.793	127 (50.4)	100 (41.8)	0.069	57 (57.0)	116 (46.4)	0.199	70 (52.6)	153 (43.5)	0.121
	65–74	182 (33.8)	19 (34.5)		87 (34.5)	86 (36.0)		30 (30.0)	95 (38.0)		38 (28.6)	134 (38.1)	
	≥ 75	98 (18.2)	8 (14.5)		38 (15.1)	53 (22.2)		13 (13.0)	39 (15.6)		25 (18.8)	65 (18.5)	
Gender	Female	159 (29.5)	16 (29.1)	1	78 (31.0)	64 (26.8)	0.358	30 (30.0)	80 (32.0)	0.813	38 (28.6)	101 (28.7)	1
	Male	380 (70.5)	39 (70.9)		174 (69.0)	175 (73.2)		70 (70.0)	170 (68.0)		95 (71.4)	251 (71.3)	
Comorbidity index	0	339 (62.9)	39 (70.9)	0.343	174 (69.0)	136 (56.9)	0.014	60 (60.0)	167 (66.8)	0.331	87 (65.4)	217 (61.6)	0.731
	1	100 (18.6)	6 (10.9)		44 (17.5)	51 (21.3)		17 (17.0)	42 (16.8)		24 (18.0)	68 (19.3)	
	2–3	100 (18.6)	10 (18.2)		34 (13.5)	52 (21.8)		23 (23.0)	41 (16.4)		22 (16.5)	67 (19.0)	
BMI	≤ 25	235 (43.6)	16 (29.1)	0.076	110 (43.7)	107 (44.8)	0.764	41 (41.0)	111 (44.4)	0.598	56 (42.1)	151 (42.9)	0.351
	25–29.9	214 (39.7)	30 (54.5)		99 (39.3)	97 (40.6)		39 (39.0)	100 (40.0)		50 (37.6)	148 (42.0)	
	≥ 30	90 (16.7)	9 (16.4)		43 (17.1)	35 (14.6)		20 (20.0)	39 (15.6)		27 (20.3)	53 (15.1)	
Smoking	Never	209 (38.9)	16 (29.6)	0.128	91 (36.1)	91 (38.6)	0.550	33 (33.0)	93 (37.5)	0.271	49 (36.8)	131 (37.5)	0.777
	Former	212 (39.5)	29 (53.7)		104 (41.3)	101 (42.8)		48 (48.0)	96 (38.7)		59 (44.4)	144 (41.3)	
	Current	116 (21.6)	9 (16.7)		57 (22.6)	44 (18.6)		19 (19.0)	59 (23.8)		25 (18.8)	74 (21.2)	
Use of NSAIDs	No	445 (83.0)	43 (79.6)	0.660	210 (83.7)	190 (80.2)	0.375	80 (80.8)	202 (81.1)	1	110 (83.3)	282 (80.8)	0.613
	Yes	91 (17.0)	11 (20.4)		41 (16.3)	47 (19.8)		19 (19.2)	47 (18.9)		22 (16.7)	67 (19.2)	
Alcohol consumption	None	59 (11.0)	4 (7.3)	0.698	30 (11.9)	23 (9.7)	0.452	10 (10.0)	32 (12.9)	0.758	15 (11.3)	39 (11.1)	0.255
	< 12	208 (38.7)	22 (40.0)		94 (37.3)	101 (42.4)		41 (41.0)	100 (40.2)		44 (33.1)	144 (41.0)	
	> 12	271 (50.4)	29 (52.7)		128 (50.8)	114 (47.9)		49 (49.0)	117 (47.0)		74 (55.6)	168 (47.9)	
Patients with available tumor marker information (n=212)													
		-	-		*n* = 116	*n* = 65		*n* = 43	*n* = 84		*n* = 60	*n* = 120	
KRAS mutation	Non-mut				83 (75.5)	45 (75.0)	1	31 (75.6)	65 (83.3)	0.441	40 (70.2)	87 (77.7)	0.379
	Mutated	-	-		27 (24.5)	15 (25.0)		10 (24.4)	13 (16.7)		17 (29.8)	25 (22.3)	
CIMP	Low/neg				109 (94.8)	58 (92.1)	0.523	41 (97.6)	78 (95.1)	-	56 (93.3)	110 (94.0)	1
	High	-	-		6 (5.2)	5 (7.9)		1 (2.4)	4 ( 4.9)		4 (6.7)	7 (6.0)	

*p* values from the chi-square or Fisher’s exact test. *Downstaging in N excluding patients with clinical N0 disease. For patients with available tumor marker information, 31 and 32 missing values are present for improvement in T and N, respectively. No tumor marker characterization performed for patients with pCR (ypT0, ypN0). No *p* value calculated if both comparison groups had < 5 patients

### Patient survival

Overall, 210 (35%) patients died among those who received neoadjuvant treatment and 342 (37%) among those who did not, 146 (70%) and 179 (52%) of whom died of rectal cancer, respectively. Among 55 patients with pCR, 4 (7%) developed recurrence of disease and died of rectal cancer, and 3 additional patients died from other causes. Among patients with downstaging in T stage and among those with downstaging in N stage, 43 (18%) and 52 (21%) presented recurrence of disease, of whom 36 and 46 died of rectal cancer, respectively. In general, presenting any type of response to neoadjuvant treatment was associated with significantly better survival (see supplementary table 1). In a complementary analysis, patients who responded to neoadjuvant treatment (downstaging in T, N, or pCR) had significantly better survival compared with patients who did not receive neoadjuvant treatment. In contrast, among patients who received neoadjuvant treatment and did not have pCR or did not present downstaging in T or N, no significant associations were observed with survival. Among patients who presented no response (neither T nor N), survival was significantly worse compared with patients who did not receive neoadjuvant treatment (see supplementary table 2).

## Discussion

In this population-based cohort study, outcomes of pCR and tumor response to neoadjuvant treatment were analyzed among resected rectal cancer patients. Among patients who received neoadjuvant treatment, almost half (49%) presented downstaging of the T stage of the tumor. This finding reflects the known benefits of neoadjuvant treatment, with reported downstaging rates between 50 and 60% [21, 22]. Among patients who presented with clinical lymph node involvement, more than half (52%) of patients were free of affected lymph nodes after neoadjuvant treatment and resection (ypN0). In this study, 9% of patients presented pCR; this finding compares with other studies that have reported rates around 10% (range 8–24%) [7, 21, 23–26].

Several molecular biomarkers to predict response to neoadjuvant treatment have been studied; however, none so far has predicted pCR or tumor response to a degree where they could be implemented in clinical practice [27, 28]. In this study, no associations between major molecular characteristics of the tumor and pCR or tumor downstaging were observed. However, among patients who presented response to nCRT and among those who presented downstaging in the number of lymph nodes, the proportion of KRAS mutations was somewhat lower. The number of patients with CIMP-hi tumors was extremely low, and thus no further analyses could be performed on this marker. Previous studies have reported conflicting results about the role of KRAS as a predictive biomarker of response to nCRT. A retrospective study of biopsies from stage II/III rectal cancer patients reported lower rates of pCR for tumors with KRAS mutations and higher rates of lymph node metastasis for tumors with both KRAS and TP53 mutations [11]. Other studies, however, have reported no such associations [29, 30]. Previous studies have suggested that there may be a difference in the response levels of KRAS mutations occurring in codons 12 and 13 (as investigated in this study) and other less frequent mutations occurring in other codons (e.g., 61 and 146) [29, 31]. Resistance to nCRT may also be influenced by intra-tumoral heterogeneity, given that different tumor sub-clones might present a variety of mutational and copy number alterations that may differentially respond to treatment [32].

Because all molecular determinations were performed on tumor specimens resected after nCRT, the association between KRAS status and pCR (ypT0–ypN0) could not be analyzed in this study. This constitutes a limitation of this analysis; however, no meaningful differences were observed in the proportion of KRAS mutations identified among patients who received nCRT and those who did not, which constitutes an interesting finding that could be of value for future research on this topic. Previous models have suggested that major genetic mutations that occur early during the tumor development are likely to persist as the tumor grows [14], indicating that the molecular alterations identified after surgical excision may still be representative of those found in the tumor before administration of nCRT. This has also been suggested by a retrospective study among 47 stage II/III rectal adenocarcinoma patients, in which genetic mutations were analyzed on tumor samples before and after nCRT and no significant differences were observed [12]. However, larger studies are needed to confirm this, given that among tumors that respond to nCRT, the original genetic mutations may no longer be detected after surgical excision. Therefore, future studies should investigate markers identified from biopsy samples to predict response to nCRT.

Inaccuracies in clinical staging might have resulted in larger observed downstaging in some cases, which might be regarded as a limitation of the study. To reduce the impact of such clinical staging inaccuracy, all patients with ambiguous cT or cN status were excluded from the analyses. Similarly, patients with TX or NX status were excluded when calculating changes in T or N stage, respectively.

Both tumor response and pCR have been identified as important predictors of survival outcomes in rectal cancer [33]. Results from a clinical trial among 400 resected rectal cancer patients who received neoadjuvant chemoradiation showed that tumor regression and ypN status were independent predictors of disease-free survival and relapse, and local recurrence, respectively [34, 35]. Other retrospective studies and meta-analyses have reported similar results [23, 24]. In this study, the survival benefit of patients who responded to nCRT was confirmed. Moreover, in exploratory analyses, non-responders had a tendency towards worse survival when compared with patients who did not receive nCRT, potentially reflecting tumors of a more aggressive nature.

Given the good prognosis of patients who respond to neoadjuvant treatment and the morbidity associated with rectal cancer surgery, the watch-and-wait approach has been proposed for patients who present cCR after nCRT [5, 36, 37]. Some studies have reported that cCR is not a perfect predictor of pCR, and therefore, patient selection should not be based solely on clinical absence of the tumor [38, 39]. Other studies have observed a trend towards better pCR and higher tumor downstaging when the time interval between nCRT and surgery is increased to between 8 and 10 weeks [26]. This indicates that waiting for a longer period after nCRT to evaluate response to treatment might be beneficial in sparing surgery for some patients. To date, no evidence from randomized clinical trials is available to recommend the watch-and-wait approach; however, several ongoing clinical trials will contribute to clarify this question (e.g., NCT02704520, NCT02008656, and NCT02945566). Because results on long-term follow-up and survival outcomes for these patients will likely take long time to be published, evidence from retrospective studies is still considered relevant to suggest potential molecular biomarkers that could be validated in larger studies.

In conclusion, in this large study with comprehensive patient characterization, molecular characteristics of rectal tumors were not significantly associated with tumor response or pCR following neoadjuvant treatment. None of the patient characteristics investigated was predictive of response to nCRT. While we found no clear indication that KRAS mutations might be associated with response to nCRT, larger studies determining molecular markers before the administration of neoadjuvant treatment are needed to investigate this more accurately. Such studies should also integrate information on other characteristic genetic changes of rectal cancer, such as APC and TP53 mutations.

## Electronic supplementary material

ESM 1 (DOCX 26 kb)
