# Supplementary material for: Response to neoadjuvant treatment among rectal cancer patients in a population-based cohort
Source: Int J Colorectal Dis. 2020 Sep 19;36(1):177–85. doi: 10.1007/s00384-020-03744-2 (PMC7782441; doi:10.1007/s00384-020-03744-2)
Supplement: Supplementary file 1 — (DOCX 26 kb) [file 384_2020_3744_MOESM1_ESM.docx]

**Supplementary material**

DACHS patients with confirmed diagnosis of rectal cancer
(n = 1726)

n = 1594

n = 1577

Included rectal cancer patients
(n = 1536)
Available tumor marker information
(n = 682)

Patients with cM1 (n = 132)

- Missing neoadjuvant treatment status (n = 9)
- No surgery performed (n = 7)
- Missing cT and cN status (n = 1)

Ambiguous cT or cN status (n = 41)

Supplementary figure 1. Patient selection criteria.

| **Supplementary table 1. Response to neoadjuvant treatment and survival among rectal cancer patients who received neoadjuvant treatment (n = 602)** | | | | | | | | | | | | | | | | |
| --- | --- | --- | --- | --- | --- | --- | --- | --- | --- | --- | --- | --- | --- | --- | --- | --- |
|  |  | **Cancer-specific survival** | | | | |  | **Relapse-free survival** | | | |  | **Overall survival** | | | |
|  |  | **N** | **Events** | **HR** | **95%CI** | **p-val** |  | **Events** | **HR** | **95%CI** | **p-val** |  | **Events** | **HR** | **95%CI** | **p-val** |
| **pCR** | No | 535 | 140 | 1 |  |  |  | 169 | 1 |  |  |  | 201 | 1 |  |  |
|  | Yes | 55 | 4 | 0.24 | 0.08-0.80 | 0.015 |  | 4 | 0.18 | 0.06-0.58 | 0.004 |  | 7 | 0.33 | 0.14-0.70 | 0.007 |
| **Downstage in T** | No | 252 | 79 | 1 |  |  |  | 93 | 1 |  |  |  | 105 | 1 |  |  |
|  | Yes | 239 | 36 | 0.55 | 0.34-0.77 | 0.001 |  | 43 | 0.50 | 0.34-0.73 | <0.001 |  | 63 | 0.62 | 0.45-0.85 | 0.004 |
| **Downstage in N** | No | 222 | 64 | 1 |  |  |  | 78 | 1 |  |  |  | 93 | 1 |  |  |
|  | Yes | 250 | 46 | 0.61 | 0.41-0.90 | 0.014 |  | 52 | 0.54 | 0.38-0.78 | <0.001 |  | 68 | 0.67 | 0.48-0.93 | 0.017 |
| **Any response** | No | 133 | 50 | 1 |  |  |  | 60 | 1 |  |  |  | 68 | 1 |  |  |
|  | Yes | 351 | 61 | 0.37 | 0.25-0.56 | <0.001 |  | 71 | 0.36 | 0.25-0.52 | <0.001 |  | 95 | 0.43 | 0.31-0.60 | <0.001 |
| *HR: adjusted Hazard ratio. pCR: pathological complete response. pCR model adjusted for: age, stage at diagnosis, adjuvant chemotherapy, BMI. Improvement in T model adjusted for age, comorbidities, stage at diagnosis and adjuvant chemotherapy. Improvement in N model adjusted for: age, adjuvant chemotherapy, stage at diagnosis, and comorbidities.* | | | | | | | | | | | | | | | | |

| **Supplementary table 2. Response or no response to neoadjuvant treatment and survival among rectal cancer patients (n = 1577).** | | | | | | | | | | | | | | | | |
| --- | --- | --- | --- | --- | --- | --- | --- | --- | --- | --- | --- | --- | --- | --- | --- | --- |
|  |  | **Cancer-specific survival** | | | | |  | **Relapse-free survival** | | | |  | **Overall survival** | | | |
|  |  | **N** | **Events** | **HR** | **95%CI** | **p-val** |  | **Events** | **HR** | **95%CI** | **p-val** |  | **Events** | **HR** | **95%CI** | **p-val** |
| **No neo** |  | 800 | 167 | 1 |  |  |  | 218 | 1 |  |  |  | 315 | 1 |  |  |
| **Neo no pCR** |  | 525 | 138 | 0.94 | 0.74-1.19 | 0.607 |  | 167 | 0.91 | 0.73-1.13 | 0.403 |  | 197 | 0.91 | 0.75-1.11 | 0.342 |
| **Neo + pCR** |  | 52 | 4 | 0.25 | 0.08-0.79 | 0.018 |  | 4 | 0.17 | 0.06-0.55 | 0.003 |  | 7 | 0.32 | 0.14-0.71 | 0.006 |
| **No neo** |  | 800 | 167 | 1 |  |  |  | 218 | 1 |  |  |  | 315 | 1 |  |  |
| **Neo no response in T** |  | 246 | 77 | 1.49 | 0.95-2.35 | 0.082 |  | 91 | 1.1 | 0.76-1.60 | 0.604 |  | 101 | 1.00 | 0.71-1.42 | 0.994 |
| **Neo + response in T** |  | 233 | 36 | 0.69 | 0.41-1.14 | 0.146 |  | 43 | 0.5 | 0.33-0.77 | 0.002 |  | 63 | 0.58 | 0.40-0.85 | 0.005 |
| **No neo** |  | 753 | 160 | 1 |  |  |  | 209 | 1 |  |  |  | 296 | 1 |  |  |
| **Neo no response in N** |  | 100 | 46 | 1.86 | 1.1-3.0 | 0.011 |  | 55 | 1.44 | 0.96-2.17 | 0.081 |  | 57 | 1.32 | 0.88-1.98 | 0.175 |
| **Neo + response in N** |  | 250 | 46 | 0.57 | 0.36-0.89 | 0.014 |  | 52 | 0.42 | 0.28-0.63 | <0.001 |  | 68 | 0.51 | 0.35-0.73 | <0.001 |
| **No neo** |  | 753 | 160 | 1 |  |  |  | 209 | 1 |  |  |  | 296 | 1 |  |  |
| **Neo no response** |  | 78 | 40 | 1.57 | 1.09-2.26 | 0.015 |  | 47 | 1.6 | 1.15-2.24 | 0.006 |  | 49 | 1.63 | 1.18-2.25 | 0.003 |
| **Neo + any response** |  | 285 | 53 | 0.49 | 0.34-0.69 | <0.001 |  | 61 | 0.45 | 0.33-0.62 | <0.001 |  | 78 | 0.58 | 0.43-0.77 | <0.001 |
| *Neo = neoadjuvant. * Improvement in N excluding patients who had clinical N0 disease. Improvement in T model excluding stage 1 patients. Improvement in N and T adjusted for age, T (N) stage at diagnosis, comorbidities and adjuvant chemotherapy. pCR model adjusted for age, stage at diagnosis, comorbidities and adjuvant chemotherapy. Any response model excluding patients who had clinical N0 disease and adjusted for age, stage at diagnosis, comorbidities and adjuvant chemotherapy.* | | | | | | | | | | | | | | | | |
